# Utilization of silver diamine fluoride by dentists in Canada: a review of the Non-Insured Health Benefits Dental Claims database

**DOI:** 10.24095/hpcdp.45.11/12.02

**Published:** 2025

**Authors:** Mohamed El Azrak, Mary F. Bertone, Anil Menon, Robert J. Schroth

**Affiliations:** 1 Department of Preventive Dental Science, Dr. Gerald Niznick College of Dentistry, Rady Faculty of Health Sciences, University of Manitoba, Winnipeg, Manitoba, Canada; 2 School of Dental Hygiene, Dr. Gerald Niznick College of Dentistry, Rady Faculty of Health Sciences, University of Manitoba, Winnipeg, Manitoba, Canada; 3 Children’s Hospital Research Institute of Manitoba, Winnipeg, Manitoba, Canada; 4 Department of Pediatrics and Child Health and Department of Community Health Sciences, Max Rady College of Medicine, Rady Faculty of Health Sciences, University of Manitoba, Winnipeg, Manitoba, Canada; 5 Shared Health Inc., Winnipeg, Manitoba, Canada

**Keywords:** oral health policy, Indigenous oral health, dental caries, prevention and control, children and youth

## Abstract

**Introduction:**

In August 2020, Indigenous Services Canada’s Non-Insured Health Benefits (NIHB) program approved reimbursement for silver diamine fluoride (SDF), a dental caries–arresting agent, for NIHB-eligible clients of all ages. We investigated the utilization of SDF for NIHB-eligible children and youth and determined trends and regional differences.

**Methods:**

The NIHB program provided data on SDF claims for children and youth (<17 years) from 1 August 2020 to 31 July 2022. We derived descriptive statistics and calculated rates of SDF application by dividing the number of children and youth with SDF claims by the number of NIHB-eligible children and youth (n=215 215).

**Results:**

There were 4158 SDF claims for 3465 children and youth (1542 in 2020–2021 and 1923 in 2021–2022, a 24.7% increase). The mean (SD) age was 7.9 (4.0) years, and 52.9% were female. General dentists made the most claims (87.1%). Manitoba had the most initial claims (19.6%), but Alberta had the highest number of follow-up claims. Nunavut (37.0/1000; 95% CI: 33.8–40.4) and Northwest Territories (20.9/1000, 95% CI: 17.2–25.1) had the highest rates of SDF claims.

**Conclusion:**

The increase in the number of SDF claims over the 2 years may indicate that more dental care providers have become aware that the NIHB program covers SDF treatment and have incorporating it into their caries treatment approaches. Still, few children and youth received follow-up SDF applications, potentially reducing the effectiveness of caries arrest.

**Highlights**

- While dental caries has historically been managed surgically, other approaches are emerging.
- In August 2020, Indigenous Services Canada’s Non-Insured Health Benefits (NIHB) program approved the use of silver diamine fluoride (SDF), an approach to managing caries that is new in Canada.
- The number of NIHB-eligible First Nations and Inuit children and youth with SDF claims increased by 24.7% between August 2020 to July 2021 and August 2021 to July 2022.
- While Ontario and the western provinces had the most claims, Nunavut and the Northwest Territories had the highest rates of claims.

## Introduction

Dental caries is one of the most prevalent chronic conditions affecting children and youth worldwide.^1^ Caries is a public health problem as it can negatively impact health and quality of life.^2^ While caries has traditionally been managed using a surgical approach, efforts to use a medical management approach are growing. Treatment with silver diamine fluoride (SDF) is a biologic approach to managing caries nonsurgically.^3^

Although SDF is a recognized caries-arresting agent, its use in Canada is relatively new. Advantage Arrest was the first SDF product approved by Health Canada, in 2017.^4^ Recent reports highlight the anticaries effects of SDF.^5^-^9^ One systematic review and meta-analysis reported that treatment with 38% SDF is effective in arresting 81% of active caries lesions in primary teeth.^10^ Current clinical guidelines indicate that SDF is the preferred nonrestorative caries management product for cavitated lesions on both primary and permanent teeth.^11^

Management with SDF could potentially reduce the need for rehabilitative dental treatment of early childhood caries (ECC) under general anaesthesia. The use of SDF has also likely grown because of the access-to-care challenges exacerbated by the COVID-19 pandemic. The major drawback of SDF is that it stains caries lesions black, an aesthetic consideration. However, a Canadian study found that parents were not particularly concerned about this staining on their children’s teeth.^12^ While the popularity of SDF use is growing, there remains a lack of consensus regarding optimal frequency of application.^13^

Indigenous Services Canada’s Non-Insured Health Benefits (NIHB) program provides health benefits to registered First Nations people and recognized Inuit for services that are not covered by other insurance or social programs such as provincial or territorial health insurance. Benefits offered through the NIHB program include dental care, vision care, prescription drugs, medical equipment and supplies, mental health counselling and medical transportation to access necessary health care not available in a claimant’s community of residence. To be eligible, NIHB program clients must be residents of Canada and either a First Nations person registered under the *Indian Act* (referred to as a “Status Indian”); an Inuk recognized by one of the four Inuit land claim organizations under their land claim agreement; or a child aged less than 2 years whose parent is an NIHB-eligible client.^14^

In August 2020, the NIHB program expanded coverage to include SDF as an eligible service under the following procedure description: “Topical application to hard tissue lesion(s) of an antimicrobial or remineralization agent (includes silver diamine fluoride).” Coverage includes three treatments in a 12-month period for those aged less than 17 years, and one treatment in a 12-month period for those aged 17 years and older, although requests for higher frequency treatments may be considered. 

The aim of this study was to investigate the utilization of SDF by dental care providers for First Nations and Inuit children and youth aged less than 17 years who receive dental benefits through the NIHB program. The objectives were to determine (1) the number of SDF claims for children and youth submitted to the NIHB program; (2) trends in the number of claims over time; (3) any regional differences in claims for SDF treatment; and (4)whether SDF treatment is provided in conjunction with traditional restorative treatments on the same day of service.

## Methods

***Ethics approval***

Ethical approval was obtained from the University of Manitoba’s Health Research Ethics Board (HS25864 H2023:049).

***Data source***

The NIHB program provided the data file containing data on SDF claims through Indigenous Services Canada via a secure data transfer portal. The provided data were for all provinces and territories except British Columbia, where the First Nations Health Authority administers health services for First Nations. As there were five or fewer claims for NIHB-eligible Inuit children and youth residing in British Columbia during the study period, these data were suppressed for reasons of privacy. Data were likewise suppressed for Prince Edward Island and Newfoundland and Labrador, which also had five or fewer claims each during the study period. The NIHB-eligible population data for these two provinces were included with the data for New Brunswick and Nova Scotia in the Atlantic region population data provided by the NIHB program.

The claims data included the following:

- Dental claim-level data for all claims paid by the NIHB program for the procedure “topical antimicrobials or remineralization agent/SDF” for children and youth with a date of service between 1 August 2020 and 31 July 2022 (first 2 years of NIHB coverage), including claims by general dentists, pediatric dentists, prosthodontists and dental hygienists.
- Dental claim-level data for all dental procedures paid by the NIHB program and claimed for the same client on the same date of service as the SDF application.
- The province or territory where the dental care provider had their practice and the provider’s field of specialty.
- Clients’ birth year and sex.
- Age group and province or territory of residence of the NIHB-eligible population.

All client and provider data were de-identified, and each client was assigned a unique identifier number. Data were stored on a password-protected server at the Children’s Hospital Research Institute of Manitoba (Winnipeg, MB).

***Procedure codes***

Because there is no specific procedure code solely for SDF for dentists (except in Quebec), we assumed that claims for “topical antimicrobials or remineralization agent/SDF” represented SDF usage by dentists (except in Quebec). We identified five procedure codes for “topical antimicrobials or remineralization agent/SDF,” of which three were for dentists: 13601 in all provinces except Quebec, 20601 for general dentists in Quebec, and 13610 for pediatric dentists in Quebec. Two codes, 00606 and 00607, were used by dental hygienists. All these codes represent, to a certain extent, the same clinical procedure.

Our analyses included procedure claims for the following: complete examinations, limited examinations, recall examinations, consultations, emergency examinations, specific examinations, intraoral radiographs, extraoral radiographs, scaling, prophylaxes, topical fluoride applications, caries trauma pain control, amalgam restorations, composite restorations, pulpotomies, stainless-steel crowns, pulpectomies, sealants, extractions, root canal treatments, general anaesthesia, and nitrous oxide and oral sedation.

Data provided by the NIHB program were complete; the only missing data referred to tooth number and tooth surface, neither of which were outcomes of interest and therefore variables in our analyses, as this information is not required when submitting SDF claims to the NIHB program.

***Analyses***

Data were reviewed and recoded in Microsoft Excel version 2404 (Microsoft Corp., Redmond, WA, US). Each client with one or more claims for SDF was assigned a row in the spreadsheet. Procedure claims were coded for each visit with a 0 to indicate that a procedure was not billed and a 1 to indicate that it was. Initial SDF claims during the study period (1August 2020 to 31 July 2022) were grouped into quarters to allow comparisons over time.

Rates of SDF claims per 1000 population (< 17 years) were calculated for each region, province or territory except for British Columbia, Prince Edward Island and Newfoundland and Labrador. The denominator was the number of NIHB clients aged less than 17 years registered in each region, province or territory; this number was provided by the NIHB program. The numerator was the number of children and youth who had a claim for SDF in each region, province or territory.

The Atlantic provinces (New Brunswick, Prince Edward Island, Nova Scotia and Newfoundland and Labrador) were grouped into the “Atlantic region” by the NIHB program as some clients are registered under “General Atlantic” and not a specific province. Because SDF claim data for Prince Edward Island and Newfoundland and Labrador were suppressed, the numerator used for the Atlantic region was the sum of children and youth who had an SDF claim in New Brunswick and Nova Scotia, whereas the denominator was the total population of NIHB-eligible children and youth (< 17 years) in the four provinces.

Data were analyzed using NCSS 2023 Statistical Software (Kaysville, UT, US). Descriptive statistics (frequencies, means and standard deviations [SD]) and 95% confidence intervals (CIs) were calculated for rates of children and youth with SDF claims. In consultation with a senior biostatistician, we compared the rates for SDF claims and the associated 95% CIs for all the provinces, territories and regions. Where 95% CIs overlapped, no differences between them were noted.

## Results

There were 4158 SDF claims for 3465 NIHB clients aged less than 17 years (Table 1). The mean (SD) age was 7.9 (4.0) years and the youngest claimant was 1 year of age. About half (52.9%) were female. Less than one-quarter (16.1%; n=558) had more than one claim.

**Table 1:** Demographic characteristics of NIHB-eligible clients (< 17 years) who received SDF treatment, and characteristics of SDF claims, 1 August 2020 to 31 July 2022, Canada

Characteristics (N = 3465)	Value
Sex of client, n (%)	
Female	1833 (52.9)
Male	1632 (47.1)
Mean (SD) age, years	7.9 (4.0)
Number of claims for SDF, n (%)	
One	2907 (83.9)
Two	436 (12.6)
Three	109 (3.2)
Four	12 (0.4)
Five	1 (0.03)
Dental care provider, n (%)	
General dentist	2831 (87.1)
Pediatric dentist	570 (16.5)
Dental hygienist	64 (1.9)
Province/territory^a^ where initial SDF claim occurred, n (%)	
Alberta	640 (18.5)
Saskatchewan	648 (18.7)
Manitoba	680 (19.6)
Ontario	605 (17.5)
Quebec	150 (4.3)
New Brunswick	112 (3.2)
Nova Scotia	11 (0.3)
Yukon	21 (0.6)
Northwest Territories	113 (3.3)
Nunavut	485 (14.0)
Other dental claim during SDF visit, n (%)	
Yes	3134 (90.5)
No	331 (9.5)
Calendar year of initial SDF claim, n (%)	
2020	539 (15.6)
2021	1757 (50.7)
2022	1169 (33.7)
Data year of initial SDF claim, n (%)	
2020/2021 (1 August 2020–31 July 2021)	1542 (44.5)
2021/2022 (1 August 2021–31 July 2022)	1923 (55.5)

**Abbreviations:** NIHB, Non-Insured Health Benefits; SD, standard deviation; SDF, silver diamine fluoride. 

^a^ Data for British Columbia, Prince Edward Island and Newfoundland and Labrador were suppressed as there were ≤ 5 claims in each of these provinces during the study period. 

Of all the provinces and territories, Manitoba had the highest proportion of initial claims for SDF (19.6%), followed by Saskatchewan (18.7%) (Table 1). Alberta and Ontario had the highest proportions of follow-up claims (Table 2). Most claims, both for the initial and follow-up visits, were submitted by general dentists (Tables 1 and 2).

**Table 2:** Characteristics of dental care providers with SDF follow-up claims for children and youth (< 17 years), 1 August 2020 to 31 July 2022, Canada

Characteristics	n (%)			
	Second SDF claim	Third SDF claim	Fourth SDF claim	Fifth SDF claim
Dental care provider				
General dentist	406 (72.9)	86 (70.5)	9 (69.2)	1 (100)
Pediatric dentist	140 (25.1)	36 (29.5)	4 (30.8)	0
Dental hygienist	11 (2.0)	0	0	0
Geographic region where SDF claims took place^a^				
Alberta	159 (28.6)	36 (29.5)	3 (23.1)	1 (100)
Saskatchewan	70 (12.6)	13 (10.7)	0	0
Manitoba	38 (6.8)	5 (4.1)	0	0
Ontario	154 (27.7)	38 (31.2)	8 (61.5)	0
Quebec	38 (6.8)	15 (12.3)	0	0
New Brunswick	37 (6.6)	7 (5.7)	1 (7.7)	0
Nova Scotia	2 (0.4)	1 (0.8)	0	0
Yukon	4 (0.7)	0	0	0
Northwest Territories	11 (2.0)	2 (1.6)	1 (7.7)	0
Nunavut	44 (7.9)	5 (4.1)	0	0
Other dental claim during the same SDF treatment visit				
Yes	402 (72.2)	81 (66.4)	11 (84.6)	0
No	155 (27.8)	41 (33.6)	2 (15.4)	1 (100)

**Abbreviation:** SDF, silver diamine fluoride. 

^a^ Data for British Columbia, Prince Edward Island and Newfoundland and Labrador were suppressed as there were ≤ 5 claims in each of these provinces during the study period.

**Figure 1:**
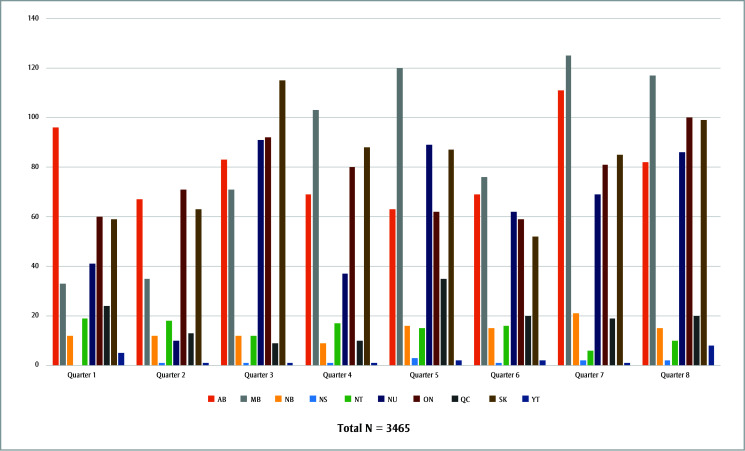
Number of initial SDF claims for children and youth (< 17 years) during each quarter from 1 August 2020 to 31 July 2022, by geographic region, Canada

**Abbreviations:** AB, Alberta; MB, Manitoba; NB, New Brunswick; NS, Nova Scotia; NT, Northwest Territories; NU, Nunavut; ON, Ontario; QC, Quebec; SDF, silver diamine fluoride; SK, Saskatchewan; YT, Yukon. 

^a^ Except British Columbia, Prince Edward Island and Newfoundland and Labrador, for which data were suppressed as there were ≤ 5 claims in each of these provinces during the study period. 

There were 1542 initial claims in the first year of the new SDF policy (1 August 2020 to 31 July 2021) and 1923 in the second year (1 August 2021 to 31 July 2022), a 24.7% increase. The number of initial claims in Quarters 1 through 8 across Canada were 349, 291, 487, 415, 492, 372, 520 and 539, respectively. (For a breakdown of claims by province and territory during each quarter, see Figure 1.) 

Rates of SDF applications based on claims submissions were calculated to adjust for the population of NIHB-eligible children and youth in each region. The highest rates of SDF claims per 1000 youth were in Nunavut (37.0; 95% CI = 33.8–40.4) and the Northwest Territories (20.9; 95% CI = 17.2–25.1). Of the provinces, Alberta (18.6; 95% CI = 17.2–20.1), Ontario (15.2; 95% CI = 14.0–16.5), Manitoba (15.1; 95% CI = 14.0–16.3) and Saskatchewan (14.4; 95% CI = 13.3–15.5) had the highest rates of SDF claims per 1000 youth (Table 3).

**Table 3:** Number and rates of SDF claims for children and youth (< 17 years), by geographic region,a 1 August 2020 to 31 July 2022, Canada

Geographic region^a^	NIHB-eligible clients (<17 years)	NIHB clients (<17 years) with SDF claim	
	Number, n	Number, n	Rates per 1000 (95% CI)
Alberta^b^	34 372	640	18.6 (17.2–20.1)
Saskatchewan^c^	45 083	648	14.4 (13.3–15.5)
Manitoba^d^	44 961	680	15.1 (14.0–16.3)
Ontario^e^	39 723	605	15.2 (14.0–16.5)
Quebec^f^	16 032	150	9.4 (7.9–11.0)
Atlantic region^g^	13 207	123	9.3 (7.7–11.1)
Yukon^h^	1292	21	16.3 (10.1–24.9)
Northwest Territories^i^	5420	113	20.9 (17.2–25.1)
Nunavut^j^	13 115	485	37.0 (33.8–40.4)

Abbreviations: CI, confidence interval; NIHB, Non-Insured Health Benefits; SDF, silver diamine fluoride. 

^a^ Except British Columbia, for which data were suppressed as there were ≤ 5 claims during the study period. 

^b^ Alberta differs from Saskatchewan, Manitoba, Ontario, Quebec, Atlantic region and Nunavut (95% CIs do not overlap). 

^c^ Saskatchewan differs from Alberta, Quebec, Atlantic region, Northwest Territories and Nunavut (95% CIs do not overlap). 

^d^ Manitoba differs from Alberta, Quebec, Atlantic region, Northwest Territories and Nunavut (95% CIs do not overlap). 

^e^ Ontario differs from Alberta, Quebec, Atlantic region, Northwest Territories and Nunavut (95% CIs do not overlap). 

^f^ Quebec differs from Alberta, Saskatchewan, Manitoba, Ontario, Northwest Territories and Nunavut (95% CIs do not overlap). 

^g ^ Atlantic region (New Brunswick, Prince Edward Island, Nova Scotia, and Newfoundland and Labrador combined) differs from Alberta, Saskatchewan, Manitoba, Ontario, Northwest Territories and Nunavut (95% CIs do not overlap). 

^h^ Yukon differs from Nunavut (95% CIs do not overlap). 

^i^ Northwest Territories differs from Saskatchewan, Manitoba, Ontario, Quebec, Atlantic region and Nunavut (95% CIs do not overlap). 

^j^ Nunavut differs from all other regions (95% CIs do not overlap). 

Most children and youth with SDF claims (90.5%) had another dental procedure at the initial SDF visit. The claims were for one or more forms of assessment and for nonrestorative, restorative or sedation procedures (Tables 1 and 4). At the time of the first SDF application, 1001 (31.9%) children and youth had a claim for a recall examination and 1669 (53.3%) for an intraoral radiograph. For nonrestorative care, 1728 (55.2%) had a claim for prophylaxis and 1539 (49.1%) for topical fluoride, while for restorative treatment, 1099 (35.1%) had a claim on the same date that SDF was applied. The most frequent restorative claims were for composites on posterior teeth (n = 741; 23.7%). Only 141 (4.5%) had a claim for nitrous oxide, 23 (3.1%) for oral sedation and 284 (9.1%) for general anaesthesia on the same day that SDF was applied (Table 4).

**Table 4:** Number and distribution of other oral health and sedation procedures claimed during the same visit as the SDF treatment claim, 1 August 2020 to 31 July 2022, Canada

Procedure claimed	n (%)			
	First visit (N = 3134)	Second visit (N = 402)	Third visit (N = 81)	Fourth visit (N = 11)
Complete examination	545 (17.4)	14 (3.5)	0	0
Limited examination	402 (12.8)	9 (2.2)	1 (1.2)	0
Recall examination	1001 (31.9)	214 (53.2)	39 (48.2)	7 (63.6)
Consultation	1 (0.0)	0	0	0
Emergency examination	87 (2.8)	6 (1.5)	2 (2.5)	0
Specific examination	302 (9.6)	29 (7.2)	7 (8.6)	1 (9.1)
Intraoral radiograph	1669 (53.3)	152 (37.8)	33 (40.7)	7 (63.6)
Extraoral radiograph	411 (13.1)	11 (2.7)	0	0
Scaling	1465 (46.8)	187 (46.5)	43 (53.1)	5 (45.5)
Prophylaxis	1728 (55.2)	220 (54.7)	40 (49.4)	7 (63.6)
Topical fluoride	1539 (49.1)	208 (51.7)	49 (60.5)	5 (45.5)
Sealant	173 (5.5)	19 (4.7)	6 (7.4)	0
Caries trauma pain control	119 (3.8)	13 (3.2)	2 (2.5)	1 (9.2)
Amalgam restoration (posterior)	43 (1.4)	8 (2.0)	1 (1.2)	0
Composite restoration (posterior)	741 (23.7)	81 (20.2)	18 (22.2)	3 (27.3)
Composite restoration (anterior)	266 (8.5)	16 (4.0)	2 (2.5)	0
Stainless-steel crown (posterior)	321 (10.3)	17 (4.2)	4 (4.9)	1 (9.1)
Stainless-steel crown (anterior)	41 (1.3)	0	1 (1.2)	0
Pulpotomy	163 (5.2)	6 (1.5)	0	2 (18.2)
Pulpectomy	18 (0.6)	0	1 (1.2)	0
Root canal	35 (1.1)	3 (0.8)	3 (3.7)	0
Extraction	420 (13.4)	26 (6.5)	5 (6.3)	0
Nitrous oxide	141 (4.5)	25 (6.2)	7 (8.6)	2 (18.2)
Oral sedation	23 (3.1)	1 (0.3)	1 (1.2)	0
General anaesthesia	284 (9.1)	6 (1.5)	1 (1.2)	0

**Abbreviation:** SDF, silver diamine fluoride. 

^a^ Except British Columbia, Prince Edward Island and Newfoundland and Labrador, for which data were suppressed as there were ≤ 5 claims in each of these provinces during the study period.

Most children and youth had another procedure performed at the same visit as the second, third and fourth follow-up claims for SDF application (Table 2). The most frequent assessment claim at the second SDF visit was for recall examinations (n=214; 53.2%). In terms of nonrestorative care, prophylaxis was claimed with 220 (54.7%) and topical fluoride with 208 (51.7%). A total of 105 (26.6%) children and youth had a restorative claim. Only 25 (6.2%) had a claim for nitrous oxide, one (0.3%) for oral sedation and six (1.5%) for general anaesthesia on the same day as the second SDF application (Table 4).

The most common assessment claim at the third SDF visit was for recall examinations (n = 39), while there were 40 claims for prophylaxis and 49 for topical fluoride. One-quarter (n = 21) were for restorative claims. Only seven claims were for nitrous oxide, one for oral sedation and one for general anaesthesia. There were seven claims for recall examination at the fourth visit and three for restorative treatment (Table 4). There were no other claims during the same visit for the one child who had a fifth claim for SDF (Table 2).

## Discussion

To our knowledge, this is the only study to investigate the uptake and trends in SDF usage by dental practitioners in Canada for First Nations and Inuit children and youth covered by the NIHB program. The NIHB program is the first national insurance plan in Canada to approve coverage for the use of SDF, and no private plans had included it as an insured service at the time of going to press. While approval of SDF coverage by many dental insurance schemes appears to be slow, the new Canadian Dental Care Plan (CDCP) includes the “topical application to hard tissue lesion(s) of an antimicrobial or remineralization agent (includes SDF)” among its covered procedures.^15^ Unlike the NIHB program, however, which covers three treatments in 12 months for children and youth, the CDCP covers two treatments in a 12-month period.

SDF can be used safely and effectively in children and youth (the youngest child to have received SDF recorded in the NIHB claims database was aged 1 year). It is used to prevent, detect and arrest caries and as a desensitizing agent.^16^ The advantages of treatment with SDF include its non-aerosol-generating ease of application, its low cost and that multiple teeth can be treated at one time. Clinical guidelines recommend application of the product for about 1 minute per carious lesion,^17^ making it suitable for people who are not able to tolerate longer restorative appointments. The main drawbacks of SDF are the black discoloration it causes on carious tooth substance, its taste and the need for follow-ups and reapplication.

The prevalence of ECC among First Nations and Inuit children is high, with reports suggesting that 85% are affected.^18^ Access to care can be a problem in Indigenous communities as there is a shortage of dentists and oral health services in many rural and remote communities.^18^,^19^ The 2014 Oral Health Survey reported that the ratio of dentists to persons was 1:2800 in Indigenous communities in the USA, which is almost half the national average of 1:1500.^18^

The use of SDF could improve access to care. Conventional treatment is expensive and can be difficult, especially for general dentists who may not have access to advanced behaviour guidance resources. Our results show that general dentists made the most claims. The ease of and atraumatic nature of SDF application can facilitate treatment by general dentists and hygienists, including in Indigenous and remote communities.

ECC often requires rehabilitative dental treatment under general anaesthesia.^20^,^21^ In Canada, children and youth from communities with high proportions of Indigenous people are more than seven times more likely to receive dental treatment under general anaesthesia than those from communities with lower Indigenous populations.^20^ Because surgical wait times may be long, SDF can be used before surgery to arrest the caries process, preventing disease progression that could complicate treatment. A study in Florida, USA, found that children on the wait-list for treatment under general anaesthesia or sedation who received SDF applications were less likely to need emergency dental care than those who did not receive SDF applications.^22^ The results from our study suggest that some dentists may have considered this approach as some clients had SDF applied prior to treatment under general anaesthesia.

SDF can also be used as part of the restorative process on a treated tooth.^23^ Suggested restorative materials include glass ionomer cement, composite resin and stainless-steel crowns.^16^,^23^ SDF used as an indirect pulp-capping material has had promising results.^24^ The NIHB claims data show that the restorative treatments most frequently provided at the same time as the SDF application were composite fillings on posterior teeth, although the data do not indicate whether the restoration was to the same tooth as the SDF application.

Studies have reported that the success of lesion arrest increases with a second application of SDF.^4^,^11^,^25^,^26^ The American Academy of Pediatric Dentistry guidelines recommends multiple applications of SDF to increase its efficacy.^17^ Our analysis shows that most children and youth only received one SDF application, which suggests that many dental care providers are unfamiliar with existing clinical guidelines for its use. The lack of access to routine dental care in remote Indigenous communities may also be a reason why multiple applications were less common.^19^ In addition, some clients may have had traditional surgical treatment of their caries at a follow-up visit.

2021 Census data show that the largest First Nations and Inuit populations are in Ontario, British Columbia, Alberta, Manitoba, Quebec and Saskatchewan, in that order.^27^ The highest number of initial claims were in these provinces (excluding British Columbia, where data on the few claims were suppressed, and Quebec). Alberta and Ontario had consistently the highest number of follow-up claims, which may be because clients in these two provinces have better access to dental care than those in other regions.

Nunavut and the Northwest Territories had the highest rates of SDF claims. Access to oral health care, community water fluoridation and nutritious foods are limited in remote regions of Canada,^28^,^29^ and dental care providers may be relying on SDF to manage caries. However, the high rates of SDF claims are most likely due to the high prevalence of caries in these regions of Canada.^28^,^29^ The high rates of SDF claims may also reflect the openness of Indigenous parents to their children receiving SDF treatment.^30^

At 9.3 and 9.4 per 1000 youth, respectively, the Atlantic region and Quebec had the lowest rates of claims for SDF. This might be because dental care providers in these provinces prefer to utilize other caries treatment modalities. Quebec, Nova Scotia and Newfoundland and Labrador have universal provincial dental-care plans for children, and dental care providers may be accustomed to tailoring treatment in accordance with the procedures these plans covered. In addition, providers may choose not to register with the NIHB program as there is a lot of overlap between the procedures the NIHB program and the universal provincial plans cover. As a result, providers in these provinces may not be aware of the updated coverage from the NIHB program.

Most initial SDF claims were made in the last two quarters of the second year of data (Quarter 7, from February to April 2022, and Quarter 8, from May to July 2022). This might be because more dentists had become aware that the NIHB program covers the service and/or had incorporated SDF into their caries treatment approaches. The fewest claims were made during the first half of the first year of data (Quarter 1, from August to October 2020, and Quarter 2, from November to January 2021) and in Quarter 6 (from November 2021 to January 2022). Quarter 1 marked the start of the NIHB program’s approval of SDF claims, and providers may not yet have been aware of the coverage. Quarters 2 and 6 were during the winter months and holiday periods, when dentists’ availability may have been limited and seeking care may be hampered by transportation challenges. It is possible that fluctuations in the numbers of COVID-19 cases may have also accounted for differences in the number of claims between each period as access to dental care may have been restricted during COVID-19 surges.

***Strengths and limitations***

Although this study is not without limitations, it is unlikely that we overestimated the use of SDF by considering all claims for “topical antimicrobials or remineralization agent/SDF” to be for SDF because the new NIHB program policy only covered SDF. In addition, the potential for coding errors in the NIHB claims database is probably low because the five codes for SDF are unique.

Not every claim included the tooth code, which is why we were unable to determine if restorative treatments were done on the teeth to which SDF was applied. The data also preclude investigating the type, number and location of the teeth that were treated with SDF. In the future, the NIHB program may want to consider implementing a means to collect information on the number of teeth treated with SDF as well as the tooth numbers and tooth surfaces. 

When calculating the number of children and youth per 1000 children and youth with a claim for SDF, we assumed that clients resided in the province or territory where they received treatment as information on their area of residence was unavailable. Clients may reside or seek treatment in a region different from where they are registered. It is also possible that jurisdictional comparisons of rates of claims for SDF may be affected by differences in the underlying age distributions in the various provinces and territories. 

Because we were only provided with the number of NIHB-eligible clients aged less than 17 years in Canada overall and in the provinces, territories and regions, we were unable to compare rates across age groups or by sex. Future studies could explore potential differences. Lastly, because the data only relate to claims, we were unable to determine whether caries lesions were successfully arrested after SDF treatment. 

## Conclusion

Our study provides valuable insights into the utilization of SDF across Canada. The data reveal a 24.7% increase in the number of registered First Nations and recognized Inuit children and youth with SDF claims from the first to the second year of the NIHB program’s expanded coverage, with most claims submitted by general dentists. While Ontario and the western provinces had the highest number of claims, Nunavut and the Northwest Territories had the highest rates of claims. We also found that few children and youth received follow-up SDF applications, potentially reducing the effectiveness of caries arrest. Further education of dental care providers may be necessary to make them aware of evidence-based clinical protocols on the use of SDF to arrest caries.

We recommend that the NIHB program consider introducing a specific code exclusively for SDF applications to facilitate further evaluation of its policy to cover SDF as an insured service. This would prevent confusion as the current billing includes topical antimicrobials or remineralization agents, not just SDF. 

## Acknowledgements

MA received funding from the Dr. Gerald Niznick College of Dentistry’s Endowment Fund. RJS holds a Canadian Institutes of Health Research Applied Public Health Chair in “Public health approaches to improve access to oral health care and oral health status for young children in Canada.”

The authors would like to express their appreciation to the NIHB program, Indigenous Services Canada, for providing the claims data to undertake this study. We would also like to thank Dr. Robert Balshaw, Centre for Healthcare Innovation, Rady Faculty of Health Sciences, University of Manitoba, for his help in our analyses. 

## Funding

This study received no external funding. The data were provided by the NIHB program through Indigenous Services Canada and accessed via a secure data transfer portal.

## Conflicts of interest

The authors’ have no conflicts of interest to declare.

## Authors’ contributions and statement

MEA: Methodology, conceptualization, data curation, formal analysis, writing—original draft, writing—review and editing.

MFB: Supervision, writing—review and editing.

AM: Supervision, writing—review and editing.

RJS: Methodology, conceptualization, data curation, supervision, project administration, writing—review and editing.

The content and views expressed in this article are those of the authors and do not necessarily reflect those of the Government of Canada.
